# Assessing universities' practices on student diversity and inclusion in higher education: The INSTADINE rubric

**DOI:** 10.12688/openreseurope.21129.2

**Published:** 2026-01-12

**Authors:** Hemendra Mistry, Maria Cruz Sancehz Gomez

**Affiliations:** 1 Department of Didactics, Organisation and Research Methods, University of Salamanca, Salamanca, Castile and León, 37008, Spain

**Keywords:** Diversity, Inclusion, Higher education, Assessment, University, INSTADINE, Rubric

## Abstract

In an effort to assist higher education institutions in becoming more inclusive of all student populations, universities are continuously reviewing their practices and policies on student diversity and inclusion. The INSTADINE rubric was developed to assist universities in assessing their practices on student diversity and inclusion in the areas of philosophy and mission, strategies, teaching, research and support services, and leadership. Each of these dimensions has a scale ranging from 1 ( *institutionalisation to some extent*) to 3 ( *full institutionalisation*) with descriptors for each score. The INSTADINE rubric has been incorporated to help universities identify their current level of attention to student diversity and inclusion in higher education, as well as to highlight areas needing further attention. The INSTADINE rubric can be available on request at mistryhemendra2015@gmail.com.

## Background

Higher education plays a vital role in fostering economic prosperity and social well-being, placing a social responsibility on universities to adopt policies that actively promote diversity and ensure equitable access to quality education. This involves a comprehensive review of curricula and educational systems to support a diverse student body and eliminate discrimination in academic environments ( Bowles & Brindle, 2017; Dill & Van Vught, 2010; Martins *et al.*, 2018). In response, many institutions have begun implementing necessary adjustments to support students from disadvantaged backgrounds- including immigrants, students from ethnic and cultural minorities, students from low socio-economic statuses, the LGBTI community, and those with special educational needs and disabilities- ensuring their full participation in the learning process ( Mistry *et al*., 2024). This commitment aligns with Sustainable Development Goal 4 (SDG 4) of the 2030 Agenda, which calls for inclusive and equitable quality education for all ( Montero *et al*., 2019; UN, 2015). In order to promote inclusive education, it is essential that universities proactively embrace diversity and implement educational policies and practices that guarantee equality and eliminate discrimination at all levels of learning ( Ainscow, 2016; Bowles & Brindle, 2017; Garcia *et al*., 2017).

The concept of diversity in education is complex and generally understood as the need to accommodate student differences and ensure equal opportunities ( Ainscow *et al*., 2007; Shah, 2008). International frameworks, including those established by the United Nations Educational, Scientific and Cultural Organisation (UNESCO) and Organisation for Economic Co-operation and Development (OECD), advocate for inclusive practices that address vulnerabilities related to socio-economic status, ethnicity, disability, gender, and other aspects ( Booth & Ainscow, 2015; OECD, 2018). For the purposes of this study, diversity includes students from immigrant, ethnic, cultural, and linguistic minorities; those from low socio-economic backgrounds; the LGBTI community; and students with special educational needs ( Mistry *et al*., 2024).

Inclusive education involves the removal of barriers and valuing individual differences to ensure that all students can participate and succeed ( Booth & Ainscow, 2011). Based on global commitments such as SDG 4, this is an evolving process that requires institutions to continuously assess and reform their policies and practices ( UNESCO, 2016). In order to ensure equal access, participation, and progress for diverse student groups, universities must adopt stratetic actions ( Garcia *et al*., 2017). Ultimately, the success of diversity and inclusion depends on institutional commitment to transforming structures, policies, and practices to support all learners equitably ( Smith, 2014; Toboso *et al*., 2019). At present, only a limited number of tools are available to evaluate the extent to which universities’ address diversity and inclusion, particularly in relation to student diversity. The development of such tools would assist universities in evaluating their current student diversity practices and identifying specific areas for improvement.

## The INSTADINE rubric

The INSTADINE rubric was developed in 2023 ( Mistry *et al*., 2024) and validated in 2025 ( Mistry & Sanchez-Gomez, 2025) as part of the USAL4EXCELLENCE MSCA COFUND fellowship project. The literature review yielded the following question: How can universities assess the progress and growth achieved in successfully addressing student diversity and their inclusion in higher education? The objective of the research was to address this question. The literature review also revealed that most of the instruments focused on overall campus-wide diversity, with student diversity being one aspect ( Mistry *et al*., 2024).

Students are the primary beneficiaries of education, and given the absence of comprehensive tools for universities to reflect on, measure, and evaluate their efforts towards student diversity and inclusion, the INSTADINE rubric has been developed to fill this gap. The rubric has been designed to function as a self-assessment tool to assist universities in determining the effectiveness of their practices, identifying areas of significant progress, and highlighting aspects that require further development. The rubric also aims to foster internal dialogue and guide universities on their path toward becoming fully inclusive.

The INSTADINE rubric is a structured tool that enables universities to evaluate their progress in addressing student diversity and inclusion in higher education. The rubric is organised into four key dimensions: (i) the university's philosophy and mission related to student diversity and inclusion (6 components); (ii) institutional strategies aimed at student diversity and inclusion (4 components); (iii) teaching, research, and support services for diverse students (6 components); and (iv) leadership and governance for fostering student diversity and inclusion (4 components). Each component comprises three levels of institutionalisation, consisting of initial efforts, moderate development, and to full integration of student diversity and inclusion practices in higher education ( Mistry *et al*., 2024).

Universities may find some items of the INSTADINE rubric difficult to address and may need to collect data using additional methods. However, by completing the rubric, universities will be able to determine the success of their efforts to promote student diversity and inclusion in higher education and identify the areas that require further attention. Universities may also use the rubric at regular intervals and as part of their phased assessment process on diversity and inclusion. The collection of data from university departments may be necessary for certain items, while others may be easily implemented by the university. In this sense, the rubric acts as a foundation for facilitating dialogue between the university and its departments as they advance towards the establishment of more just and inclusive environment for all students.

## INSTADINE rubric development process

The INSTADINE rubric was developed as part of the USAL4EXCELLENCE MSCA COFUND research fellowship project with the aim of assisting universities in the self-assessment of their policies and practices on student diversity and inclusion in higher education. A review of the academic literature was conducted to establish the framework for the rubric's structure, design, and layout of the rubric in line with its objective of evaluating university practices and policies related to student diversity and inclusion in higher education. A self-assessment rubric was developed based on a set of four dimensions and 20 indicators, which constitute categories. The quality of the rubric was ensured by considering three criteria: detailed descriptions of each dimension, indicators, and scoring procedures ( Dogan & Uluman, 2017; Gatica-Lara & Uribarren-Berrueta, 2013).

To promote inclusive education, a rubric applicable to all students, including those excluded due to their background was created. An attempt was made to construct items applicable to all categories of diversity, rather than focusing specifically on one particular diverse category. The rubric addresses the policies and practices on student diversity that encourage or require attention for their inclusion in higher education.

The rubric was designed to serve as a valuable addition to existing assessment frameworks, such as the Equity Scorecard ( Bensimon, 2004) and the Inclusive Excellence Scorecard ( Williams *et al.*, 2005). In the development of the new rubric, Furco’s (1999) self-assessment model for institutionalising service learning in higher education has been taken into account, as well as different comprehensive assessment tools, including the New England Resource Centre for Higher Education (NERCHE) rubric (2016), the PULSE DEI rubric ( Brancaccio-Taras *et al*., 2022), adapted rubric developed at institutions such as the University of California Berkeley, the University of Colorado Denver, and the University of Michigan. However, despite the fact that these rubrics address various aspects of diversity and inclusion, they do not focus specifically on student diversity and inclusion or have a limited focus, such as race ( Brancaccio-Taras *et al*., 2022). Following the NERCHE rubric as a basis, the Institutionalisation of Attention to Diversity and Inclusive Education (INSTADINE) rubric was thus developed to help assess universities' efforts to address student diversity and inclusion in higher education. The rubric focuses on the university's mission and philosophy, institutional strategies on student diversity, inclusion and teaching-learning, and leadership.

The initial draft of the INSTADINE rubric was referred to a panel of 15 experts in the fields of equality, diversity and inclusion for their judgement on the items and comments to further improve the items in the rubric. The recommendations made by the experts resulted in the revision of the rubric items with lower validity values. Following the incorporation of the suggestions, an updated draft of the rubric was field-tested in 32 universities across four European countries. The final version of the INSTADINE rubric can be available on request at mistryhemendra2015@gmail.com for further use.

## Structure and purpose of the INSTADINE rubric

As previously mentioned, the INSTADINE rubric encompasses four key dimensions: (i) mission and philosophy, (ii) institutional strategies aimed at student diversity and inclusion, (iii) inclusive teaching and learning, research, and student support services, and (iv) leadership. Due to the length of the rubric, it was not feasible to include components related to accessibility, assessment, and well-being. In addition, during the validation process of the rubric, the experts rated the four selected dimensions very positively. Each of the four dimensions was precisely defined, and the rubric was further developed using an application guide that contextualised the objective of the study, the dimensions and indicators for measuring student diversity and inclusion, and the purpose, use, and application instructions of the rubric. Each dimension of the rubric comprises four to six indicators, each targeting key aspects relevant to that dimension. Each indicator includes a detailed description of three scenarios that were established regarding student diversity and inclusion in higher education: institutionalisation to some extent, an average level of institutionalisation, and total institutionalisation. Each indicator is assigned a score on a scale ranging from 1 to 3: *Beginning* = 1, *Developing* = 2, and *Accomplished* = 3. Each performance level is accompanied by detailed descriptors that align with the context of the respective indicator. These descriptors assist universities in evaluating the current status of their efforts on student diversity and inclusion (see Table 1A).

**Table 1A.** INSTADINE rubric structure.

(A) University philosophy and mission on student diversity and inclusion in higher education		
(A1) Definition of student diversity and inclusion		Self-assigned score
**Context:** This component examines whether the university has its definition of student diversity and inclusion that addresses most of the aspects of student diversity and inclusion in higher education. The definition of student diversity and inclusion in higher education is crucial for the creation of a learning environment that values all individuals and fosters the success of all students, thereby contributing to a more just and inclusive society ( Dari, 2023; Hubbard & Gawthorpe, n.d.).		
A1.1	The university has an operational definition of student diversity and inclusion but it only addresses limited aspects of student diversity (such as race, culture and disability).	1
A1.2	The university has an operational definition of student diversity and inclusion, but its implementation varies and is inconsistent across different student diversities.	2
A1.3	The university has a universally accepted definition of student diversity and inclusion that addresses most of the aspects of student diversity and inclusion in higher education.	3
*Comments (if any):*		

*Each dimension of the rubric contains an explanation of the dimension and scoring categories, 1 to 3.*

The INSTADINE rubric requires universities to determine a consensus score for each dimension of the rubric and an overall average score for the entire rubric. The university can distribute the rubric to each department, facilitate discussions on its components to reach a consensus, and collaboratively determine an overall university score. In order to arrive at a final score, it will be necessary to collect data from departments and conversations within departments and at the university level.

## INSTADINE rubric components

The following section provides a brief overview of the components within each of the four INSTADINE dimensions, along with the rationale for their inclusion in the rubric. These four dimensions are as follows: (A) the university philosophy and mission on student diversity and inclusion in higher education; (B) institutional strategies focused on promoting student diversity and inclusion in higher education; (C) Inclusive teaching, research, and student support services; and (D) leadership in advancing student diversity and inclusion. Table 1B presents a summary of the components of the INSTADINE rubric.

**Table 1B.** INSTADINE rubric dimensions and components.

INSTADINE Rubric	
A	University philosophy and mission on student diversity and inclusion in higher education
A1	Definition of student diversity and inclusion-
A2	Strategic planning for student diversity and inclusion-
A3	Alignment with the mission of the university-
A4	Relationship between the university’s organisational structure and student diversity and inclusion-
A5	Visibility of student diversity on the university campus-
A6	Incorporation of student diversity and inclusion into accreditation processes-
B	Institutional strategies aimed at student diversity and inclusion in higher education
B1	Awareness raising and dissemination of information on student diversity and inclusion-
B2	Collaboration with diversity organisations-
B3	Equal partnership opportunities for students from diverse backgrounds-
B4	Learning achievement of students with diversity-
C	Inclusive teaching, research, and support services for students
C1	Faculty knowledge and awareness of student diversity and inclusion-
C2	Curriculum and its adaptations-
C3	Inclusive teaching-learning methods and approaches-
C4	Inclusive teaching-learning resources-
C5	Research on student diversity and inclusion-
C6	Support services for student diversity and inclusion in higher education-
D	Leadership for student diversity and inclusion
D1	Diversity and inclusion coordination-
D2	Recognition of student diversity and inclusion by the policy-making bodies-
D3	Professional development opportunities for faculty and staff-
D4	Funding of activities related to student diversity and inclusion-

*The INSTADINE rubric contains four dimensions (A to D). Each dimension has four to six components.*

### (A) University philosophy and mission on student diversity and inclusion in higher education

The six components of the university's philosophy and mission include the definition of student diversity and inclusion, strategic planning for student diversity and inclusion, alignment with the university's mission, linkage between the university’s organisational structure and student diversity and inclusion, noticeable student diversity on campus, and incorporation of student diversity and inclusion into accreditation processes.

***(A1) Definition of student diversity and inclusion***

This component examines whether the university has its definition of student diversity and inclusion that addresses most of the aspects of student diversity and inclusion in higher education. The definition of student diversity and inclusion in higher education is crucial for the creation of a learning environment that values all individuals and fosters the success of all students, thereby contributing to a more just and inclusive society ( Dari, 2023; Hubbard & Gawthorpe, n.d.).

***(A2) Strategic planning for student diversity and inclusion***

According to the Society for College and University Planning (n.d.), student diversity and inclusion planning in higher education involves intentionally building a diverse student community; empowering all members to reach their full potential by dismantling systemic barriers and addressing historical inequities; and cultivating a campus culture that values, respects, and embraces difference. The rubric scrutinises the university's strategic plans, encompassing both immediate and long-term objectives, with the aim of promoting student diversity and inclusion on campus, ensuring that all students, including those from diverse backgrounds, experience a sense of worth and inclusion in higher education.

***(A3) Alignment with the mission of the university***

Diversity and inclusion values are instrumental to fulfilling the strategic missions of universities ( Sweet, n.d.). An analysis of the alignment of student diversity and inclusion with the university's mission is included in the rubric, as universities are required to be inclusive of all students, so student diversity and inclusion should be of primary concern and reflected in the official mission of the university.

***(A4) Relationship between the university’s organisational structure with student diversity and inclusion***

The alignment of student diversity with the university’s organisational structure involves the integration of it into all institutional elements, including the curriculum and admission processes. This approach is essential to the creation of an inclusive and equitable educational environment that supports and benefits all students, particularly those from diverse backgrounds. This component was included in the rubric because it addresses the importance of linking the university's organisational structure with diverse student bodies purposefully so that the diverse students feel represented and their voices can be heard.

***(A5) Visibility of student diversity on the university campus***

Universities can be considered diverse and inclusive for all students when visible diversity is present on their campuses. Diversity on university campuses encourages self-reflection, produces better results, and promotes creative thinking ( Purdue Global, 2023). The representation of diverse students on university campuses is vital for enhancing learning experiences, fostering social and intellectual growth, and preparing students for a diverse and globalized world ( Shorelight Team, 2023). This component examines whether the university campus represents various diverse groups and whether it has developed mechanisms for accessibility and retention that reflect the diversity of the student population.

***(A6) Incorporation of student diversity and inclusion into accreditation processes***

This component emphasises the incorporation of student diversity and inclusion into accreditation processes. The integration of student diversity and inclusion into accreditation processes is imperative for ensuring that institutions effectively serve all learners and prepare them for a diverse society ( NASPAA, n.d.). In order to achieve this, it is essential to engage students in the accreditation process, integrate diverse perspectives within curricula and teaching practices, and adopt inclusive and equitable assessment strategies ( Belhadia, 2025). Universities can use this rubric component to evaluate both their internal and external accreditation processes, taking into account student diversity and inclusion as they work toward becoming more diverse and inclusive universities.

### (B) Institutional strategies focused on promoting student diversity and inclusion in higher education

The four components of assessment explored by universities include diversity awareness and information dissemination, collaboration with diversity organisations, provision of equal partnership opportunities for students from diverse backgrounds, and assessment of the learning achievements of students from diverse backgrounds.

***(B1) Awareness raising and dissemination of information on student diversity***

This component was included to assess the university's efforts to develop awareness programmes and to disseminate information, in both online and offline media, pertaining to student diversity and inclusion to the university community, including faculty, staff and students. In addition to the frequent organisation of awareness-raising programmes for faculty, staff and students, the adoption of consistent actions aimed at student diversity and inclusion helps the university community to become more familiar with student diversity and inclusion in higher education.

***(B2) Collaboration with diversity organisations***

This component enables universities to consider collaborations with governmental and non-governmental organisations to assist and address the diverse needs of the student population on campus. This type of collaboration is important to obtain support that addresses the diverse personal and educational needs of students, which makes their higher education experience more accessible.

***(B3) Equal partnership opportunities for students from diverse backgrounds***

Students report feeling more included when they are encouraged and offered equal participation not just in curricular but also in co-curricular activities, leading to their increased involvement. The provision of equal partnership opportunities for students from diverse backgrounds is key to the creation of a more equitable and inclusive educational environment. It promotes a sense of belonging, encourages cross-cultural understanding, and prepares students for a diverse and interconnected world ( Dari, 2023). This component examines whether students from diverse backgrounds are encouraged to participate in curricular and co-curricular activities as part of their equal partnership.

***(B4) Learning achievement of students with diversity***

The rubric includes this criterion to enable universities to evaluate the learning outcomes of the students with a focus on their diversity and inclusion. By examining the academic performance of diverse student populations universities can identify disparities in learning and implement tailored intervention to improve learning outcomes. Each university must disaggregate student performance data to identify any disparities in outcomes that require attention.

### C. Inclusive teaching, research, and student support services for

The six components of institutional strategies for teaching, research, and support services focus on faculty members' knowledge and understanding of student diversity and inclusion, curriculum and its adaptation, teaching-learning methods and approaches, teaching-learning resources, research on student diversity and inclusion, and supporting services available for diverse students.

***(C1) Faculty knowledge and awareness of student diversity and inclusion***

The development of knowledge about the inclusion of students from diverse backgrounds requires an understanding of relevant terminology, the different types of student diversity, and the specific needs of these students. This can help teachers to consider diversity and inclusion in teaching-learning and assessment activities. The inclusion of this component was intended to encourage universities to reflect on their faculty members' knowledge and understanding of student diversity and inclusion in higher education. Teachers' understanding of student diversity can help them to be more effective in developing inclusive lesson plans ( Østerlie & Jusslin, 2024).

***(C2) Curriculum and curriculum adaptation***

Curriculum adaptation involves differentiation to meet the needs of all students ( Julka, 2016). Such adaptation is crucial in an inclusive classroom to ensure that all student, regardless of their individual learning needs, receives appropriate modifications to content, teaching methods, assessments, and learning environments. This, in turn, creates a more equitable and engaging learning experience for all. This component helps universities evaluate the extent to which the curriculum is accessible to all students, regardless of their diverse needs, and to identify which parts of the curriculum require adaptation to ensure the inclusion of all students, including those from diverse backgrounds.

***(C3) Teaching-learning methods and approaches***

Inclusive teaching and learning practices have the potential to engage all students and contribute positively to outcomes such as student retention, academic achievement, and progression ( Thomas & May, 2010). This component was included in the rubric to encourage universities to reflect on the efficiency with which they are incorporating inclusive methods and approaches in their teaching, in order to meet the diverse learning needs of their student population.

***(C4) Teaching-learning resources***

To implement inclusive education effectively, it is essential to equip teachers with the necessary resources and support, while also providing students with inclusive learning materials that foster a sense of belonging and ensure that they feel valued and included within the learning community ( College of Education, 2024). This component facilitates universities in assessing the teaching and learning resources, including teaching-learning centres and mentoring programmes, available on university campuses to support diverse learning experiences and ensure the full inclusion of students in the teaching-learning process.

***(C5) Research on student diversity and inclusion***

Research on student diversity and inclusion has the potential to inform best practices, improve student outcomes, and promote more equitable and inclusive learning environment ( Bow, 2024; Purdue Global, 2023). Research can also facilitate the identification of gaps and address systematic biases and inequalities that may disadvantage certain student groups ( Advance HE, 2018). This component was therefore incorporated into the rubric to encourage universities to reflect on their efforts, obtain feedback and identify the policy-practice gaps that affect the accommodating student diversity and full inclusion.

***(C6) Support services for diverse students***

Support services contribute to the creation of an inclusive environment in which students from diverse backgrounds can thrive ( Halpin, n.d.). This criterion enables universities to evaluate their supporting services which cater to the diverse needs of students, including accessibility, assistive technology, and equal opportunity units, in order to make the higher education experience accessible for all students.

### D. Leadership in advancing student diversity and inclusion

The four components of leadership address the diversity and inclusion coordination, the recognition of student diversity and inclusion by the policy-making bodies, the professional development opportunities for faculty and staff, and the funding for activities related to student diversity and inclusion.

***(D1) Diversity and inclusion coordination***

The establishment of a formal entity to support diversity and inclusion practices within the university is crucial for the effectively coordination, promotion and implemention of diversity and inclusion policies and practices, and the tracking of their progress. The inclusion of this component in the rubric was intended to assist universities in evaluating the implementation and coordination of their diversity and inclusion initiatives.

***(D2) Recognition of student diversity and inclusion by policy-making bodies***

The component stipulate that universities must acknowledge student diversity and inclusion, thereby ensuring that students from diverse backgrounds are fully included in higher education by addressing relevant policies established by the policy-making bodies. This is of particular significance, given that equality and inclusion have emerged as key policy priorities due to the advancement of access and quality in the higher education system worldwide ( UNESCO, 2023).

***(D3) Professional development opportunities for faculty and staff***

This component was incorporated into the rubric because acquiring new skills, lifelong learning and the refinement of understanding of concepts related to diversity and inclusion are important for practising inclusion and meeting the diverse needs of students in higher education ( McEwen *et al*., 2025). This component enables universities to evaluate the scope of professional development opportunities offered to faculty and staff, including access to electronic and print resources on student diversity and inclusion, as well as training programmes that address relevant policies, provisions, and practices that promote student equality, diversity and inclusion in higher education ( AAC&U, 2022; Centre for the Study of Social Policy, 2019). A lack of knowledge to address the diverse needs of students can lead to ineffective inclusion practices.

***(D4) Funding of activities related to student diversity and inclusion***

In addition to ensuring accessibility, securing sufficient funding for student diversity and inclusion initiatives in higher education is essential to guarantee that students from diverse backgrounds can fully benefits and succeed in higher education without any financial barriers ( Young Invincibles, 2024). Insufficient funding can significantly hinder universities’ efforts to sustain student diversity and foster inclusive environments on their campus ( EUA, 2019). This component enables universities to consider the promotion of activities related to student diversity and inclusion through the allocation of sufficient funds, thereby ensuring the sustained implementation of such activities on campus.

## Rubric limitations

It should be noted the INSTADINE rubric has different limitations. The focus of the INSTADINE rubric was a deliberate design decision, modelled after the approach used by NERCHE. Despite the efforts to ensure the rubric is inclusive of all students, including those from diverse backgrounds, some items may require revisions to focus on specific marginalised groups such as people with disabilities. However, the items included in the INSTADINE rubric promote inclusivity and awareness of diverse identities, as well as diversity and inclusion in general. In order to ensure that their efforts address all diverse categories or a specific diverse category, if the case may be, universities may revise or add items.

In addition, the rubric did not cover components or any items related to well-being and participation, employability and mobility, and student-centered learning. Although not exhaustive, the INSTADINE rubric is intended to help universities initiate a meaningful evaluation of their current efforts in student diversity and inclusion in higher education and guide them towards further improvement.

Finally, another limitation of the rubric is that universities require inter-departmental deliberations, which may hinder consensus. However, given the wider use of the INSTADINE rubric across universities, it is not a static instrument and will undergo revisions over time.

## Future prospects

The researchers intent to further development of the rubric by collecting data from all Spanish universities to assess the efforts on student diversity and inclusion in higher education in Spain. Furthermore, the rubric intended to be developed further by the addition of the dimensions such as well-being and participation, employability and mobility, student performance and student-centered learning. This will facilitate the determination of the state of student diversity and inclusion in higher education to better serve universities and help in improving their efforts in this area.

The use of the INSTADINE rubric can serve as a valuable tool for the evaluation of universities policies on student diversity and inclusion, thereby facilitating the identification of practices that require further attention to ensure the inclusion of all students in higher education. It also supports efforts to increase the participation of diverse student groups. The use of the rubric for self-assessment purposes enables universities to monitor their progress and identify areas that require further attention. Universities that rate themselves as effective in promoting student diversity and inclusion can act as role models for other institutions both nationally and internationally.

## Ethics and consent

This study was a part of an MSCA Cofund research project on the institutionalisation of student diversity and inclusion in higher education, approved by the Research Ethics Committee of the University of Salamanca (protocol code 857, January 25, 2023). However, this method study focused on developing a rubric and did not involve collecting or analysing data; therefore, separate ethical approval and consent were not required.

## Declarations

### Data availability statement

The study focusses on developing a rubric and therefore does not include any primary, secondary, or supplementary data or analysis.
