# Supplementary material for: Assessing universities' practices on student diversity and inclusion in higher education: The INSTADINE rubric
Source: Open Res Eur. 2026 Jan 12;5:263. Originally published 2025 Aug 28. [Version 2] doi: 10.12688/openreseurope.21129.2 (PMC12824476; doi:10.12688/openreseurope.21129.2)
Supplement: Peer review history

# Reviewer report: Reviewer response for version 2

**DOI:** 10.21956/openreseurope.24037.r67970

**Published:** 2026-01-21

**Authors:** B. Morgado Camacho

**Affiliations:** 1 Developmental and Educational Psychology, University of Seville, Seville, Spain

I have reviewed the paper and I consider approve it for indexing.

Are sufficient details provided to allow replication of the method development and its use by others?

Partly

Is the description of the method technically sound?

Yes

Are the conclusions about the method and its performance adequately supported by the findings presented in the article?

Yes

Is the rationale for developing the new method (or application) clearly explained

Yes

If any results are presented, are all the source data underlying the results available to ensure full reproducibility?

No source data required

Reviewer Expertise:

My experice area is about incusive education in Higher Education; inclusive metthodologies; inclusive pedagogy; barriers and aims to facilitate success of students with disability

I confirm that I have read this submission and believe that I have an appropriate level of expertise to confirm that it is of an acceptable scientific standard.

# Reviewer report: Reviewer response for version 2

**DOI:** 10.21956/openreseurope.24037.r67969

**Published:** 2026-01-13

**Authors:** Martin Skutil

**Affiliations:** 1 Department of Education, Charles University, Faculty of Arts, Prague, Czech Republic

The issues raised in the original review have been clarified to a certain extent. For this reason, I have no further comments or recommendations.

Are sufficient details provided to allow replication of the method development and its use by others?

Partly

Is the description of the method technically sound?

Yes

Are the conclusions about the method and its performance adequately supported by the findings presented in the article?

Partly

Is the rationale for developing the new method (or application) clearly explained

Yes

If any results are presented, are all the source data underlying the results available to ensure full reproducibility?

No source data required

Reviewer Expertise:

education; inclusive education; teacher training

I confirm that I have read this submission and believe that I have an appropriate level of expertise to confirm that it is of an acceptable scientific standard.

# Reviewer report: Reviewer response for version 1

**DOI:** 10.21956/openreseurope.22855.r59501

**Published:** 2025-10-17

**Authors:** B. Morgado Camacho

**Affiliations:** 1 Developmental and Educational Psychology, University of Seville, Seville, Spain

The paper presents an assessment scale to help universities self-assess their diversity and inclusion efforts. The scale consists of four dimensions, each of which includes four to six indicators. The study is very interesting and adequately describes the assessment tool or scale. However, more detailed information on the procedure for collecting the information needs to be included. This is so that all universities that want to use the scale to assess the status of diversity and inclusion at their university know and are clear about the steps to follow.

 Likewise, they should describe how to proceed with interdepartmental dialogue to carry out the evaluation of each university (how many departments, who should participate, number of representatives from each department, etc.).

Are sufficient details provided to allow replication of the method development and its use by others?

Partly

Is the description of the method technically sound?

Yes

Are the conclusions about the method and its performance adequately supported by the findings presented in the article?

Yes

Is the rationale for developing the new method (or application) clearly explained

Yes

If any results are presented, are all the source data underlying the results available to ensure full reproducibility?

No source data required

Reviewer Expertise:

My experice area is about incusive education in Higher Education; inclusive metthodologies; inclusive pedagogy; barriers and aims to facilitate success of students with disability

I confirm that I have read this submission and believe that I have an appropriate level of expertise to confirm that it is of an acceptable scientific standard, however I have significant reservations, as outlined above.

# Response

**Published:** 2025-11-04

**Authors:** Hemendra Mistry

**Affiliations:** The Maharaja Sayajirao University of Baroda, India

Thank you for the review and providing your valuable comments. Regarding the description of process with interdepartmental dialogue to carry out the evaluation of the university (how many departments, who should participate, the number of representatives from each department, etc.), the vice-rector, EDI coordinator, or person in charge of equality, diversity and inclusion (EDI) at the university level may contact the department-level staff engaged with EDI-related activities and gather the required information on particular aspects of the rubric which require interdepartmental deliberations. In addition, to examine available records, facilities, reports, and resources, they may conduct interviews or focus group discussions with the respective staff and verify before assigning the scores and add comments (if required).

 As the current paper solely focused on the development of the rubric, all the details of the rubric is not covered. However, the full rubric manual is available open access on the University of Salamanca website (already mentioned in the abstract), which covers all the details about the rubric components and how to use the rubric.

# Reviewer report: Reviewer response for version 1

**DOI:** 10.21956/openreseurope.22855.r59503

**Published:** 2025-09-10

**Authors:** Martin Skutil

**Affiliations:** 1 Department of Education, Charles University, Faculty of Arts, Prague, Czech Republic

The submitted article constitutes an interesting and enriching contribution to the ongoing discourse on the nature of inclusive approaches within higher education. The situational context is clearly delineated and sufficiently substantiated, drawing on current and relevant scholarly literature. On the other hand, particularly in the introductory sections, the text exhibits a certain redundancy through repeated references to the INSTADINE rubric. While it is understandable that the authors seek to underscore the importance of this rubric, such duplication appears superfluous.

 For a more profound understanding of its significance, a more explicit justification of the selection and construction of the individual categories under examination would be desirable. The rationale provided remains largely descriptive, stating outcomes rather than explicating the actual process of development and selection. This limitation is further reflected in the fact that the manuscript concentrates on the construction of the instrument, yet does not include a detailed analysis of the results from a pilot study nor a statistical evaluation of its reliability and validity. The absence of pilot testing and its systematic evaluation undermines the scientific credibility of the instrument, as its generalizability cannot be assessed without empirical data.

 Several further questions emerge from the text, most notably whether a more comprehensive instrument should have been developed so that universities would not be required to supplement it with their own categories. Was this an intentional decision by the authors in order to take into account local or national contexts, or was it meant to enable individual countries or regions to adapt the instrument according to their particular needs? Even within Spain, notable regional differences exist, not to mention the broader cultural distinctions across Europe and indeed the global context. These aspects would benefit from more thorough elaboration.

 Another area of ambiguity concerns the actual use of the instrument within a single university. The INSTADINE rubric appears to be designed primarily for the macro-level of university institutions, as exemplified by categories such as the vision and mission of the university. At the same time, however, it inquires into teaching methods situated within the micro-level of the educational process—an area to which institutions themselves may be unable to respond adequately, apart from outlining their conceptual orientation, which does not necessarily reflect lived reality. It would therefore be valuable to provide a clearer explanation of the intended application of this instrument in practice, including a critical reflection on the potential risks associated with its implementation within the domain of tertiary education. These aspects should be further elaborated in the text, and analytical conclusions should be incorporated into the article.

 After minor revisions, I recommend the article for indexing.

Are sufficient details provided to allow replication of the method development and its use by others?

Partly

Is the description of the method technically sound?

Yes

Are the conclusions about the method and its performance adequately supported by the findings presented in the article?

Partly

Is the rationale for developing the new method (or application) clearly explained

Yes

If any results are presented, are all the source data underlying the results available to ensure full reproducibility?

No source data required

Reviewer Expertise:

education; inclusive education; teacher training

I confirm that I have read this submission and believe that I have an appropriate level of expertise to confirm that it is of an acceptable scientific standard, however I have significant reservations, as outlined above.

# Response

**Published:** 2025-11-04

**Authors:** Hemendra Mistry

**Affiliations:** The Maharaja Sayajirao University of Baroda, India

Thank you for your valuable comments. However, the current article is solely focused on the development of a rubric not the validation or pilot testing. The rubric has been pilot tested in four European countries, which will be discussed in separate articles that are set to be published soon and will be available open access. The goal was to develop a comprehensive rubric; however, initial deliberation with the experts in the fields of equality, diversity and inclusion, and literature review resulted in the four main dimensions. 15 experts from different countries evaluated the rubric (see in acknowledgement section) and found the rubric as valid tool. Based on the qualitative inputs of the experts and considering the length of the rubric, it was not feasible to cover many dimensions, which is already highlighted in the limitation section. As As mentioned previously, the results of the experts’ validation and pilot testing will clarify further all the points raised by the reviewers. The rubric is intended to be responded by the vice-rector, EDI coordinator, or person in charge of equality, diversity and inclusion (EDI) at the university level. However, s/he may contact the department-level staff engaged with EDI-related activities and gather the required information on the micro-level aspects of the rubric, which require interdepartmental deliberations. In addition, to examine available records, facilities, reports, and resources, they may conduct interviews or focus group discussions with the respective staff and students and verify before assigning the scores and add comments (if required). As the current paper solely focused on the development of the rubric, all the details of the rubric are not covered. However, the complete rubric manual is available for open access on the University of Salamanca website, as mentioned in the abstract, and it includes all the details about the rubric components and instructions for using the rubric.
